# Investigating the mechanism of photoisomerization in jellyfish rhodopsin with the counterion at an atypical position

**DOI:** 10.1016/j.jbc.2023.104726

**Published:** 2023-04-23

**Authors:** Shino Inukai, Kota Katayama, Mitsumasa Koyanagi, Akihisa Terakita, Hideki Kandori

**Affiliations:** 1 Department of Life Science and Applied Chemistry, Nagoya Institute of Technology, Nagoya, Japan; 2 OptoBioTechnology Research Center, Nagoya Institute of Technology, Nagoya, Japan; 3 PRESTO, Japan Science and Technology Agency, Kawaguchi, Saitama, Japan; 4 Department of Biology, Graduate School of Science, Osaka Metropolitan University, Osaka, Japan

**Keywords:** animal rhodopsin, jellyfish, counterion, isomerization, FTIR

## Abstract

The position of the counterion in animal rhodopsins plays a crucial role in maintaining visible light sensitivity and facilitating the photoisomerization of their retinal chromophore. The counterion displacement is thought to be closely related to the evolution of rhodopsins, with different positions found in invertebrates and vertebrates. Interestingly, box jellyfish rhodopsin (JelRh) acquired the counterion in transmembrane 2 independently. This is a unique feature, as in most animal rhodopsins, the counterion is found in a different location. In this study, we used Fourier Transform Infrared spectroscopy to examine the structural changes that occur in the early photointermediate state of JelRh. We aimed to determine whether the photochemistry of JelRh is similar to that of other animal rhodopsins by comparing its spectra to those of vertebrate bovine rhodopsin (BovRh) and invertebrate squid rhodopsin (SquRh). We observed that the N-D stretching band of the retinal Schiff base was similar to that of BovRh, indicating the interaction between the Schiff base and the counterion is similar in both rhodopsins, despite their different counterion positions. Furthermore, we found that the chemical structure of the retinal in JelRh is similar to that in BovRh, including the changes in the hydrogen-out-of-plane band that indicates a retinal distortion. Overall, the protein conformational changes induced by the photoisomerization of JelRh yielded spectra that resemble an intermediate between BovRh and SquRh, suggesting a unique spectral property of JelRh, and making it the only animal rhodopsin with a counterion in TM2 and an ability to activate G_s_ protein.

Most animal rhodopsins are light-sensitive G protein–coupled receptors (GPCRs) that absorb photons through a chromophore molecule called 11-*cis*-retinal, which covalently binds to a protein moiety through a Schiff base linkage with a lysine residue (1, 2, 3, 4). These rhodopsins share common structural elements with GPCRs, featuring seven transmembrane helices, and function as light sensors for various physiological events such as vision and regulation of biological rhythms (5). Upon absorption of light, 11-*cis*-retinal is photoisomerized to all-*trans*-retinal, and animal rhodopsins undergo a series of conformational changes to form the signaling-competent Meta-II intermediate state, activating G protein.

The protonation state of the retinal Schiff base is a well-characterized factor that determines the absorbance spectra of rhodopsins. The unprotonated retinal Schiff base absorbs in the UV region, while the protonated retinal Schiff base covers the entire visible light spectrum. Additionally, the protonated retinal Schiff base allows for extremely fast "*cis-trans*" isomerization through an almost barrierless potential energy surface of the S1 excited state (6). The Schiff base proton is stabilized by the negative charge of an amino acid residue acting as the counterion (Fig. 1*A*). In vertebrate rhodopsins, the Schiff base counterion has been identified as Glu113 on transmembrane 3 (TM3) (7, 8, 9). Structurally, Glu113 is located near the Schiff base, suggesting that it forms a direct salt bridge (10, 11), which is consistent with the FTIR results (Fig. 1*B*) (12). In contrast, previous mutational analysis has revealed that Glu181, which is situated in the second extracellular loop (ECL2) connecting TM4 and TM5, acts as the counterion in invertebrate rhodopsins (13, 14, 15). This has implied that the counterion has been switched from Glu181 to Glu113 during the evolution of vertebrate rhodopsins (Fig. 1*B*) (13, 14, 15).

**Figure 1:**
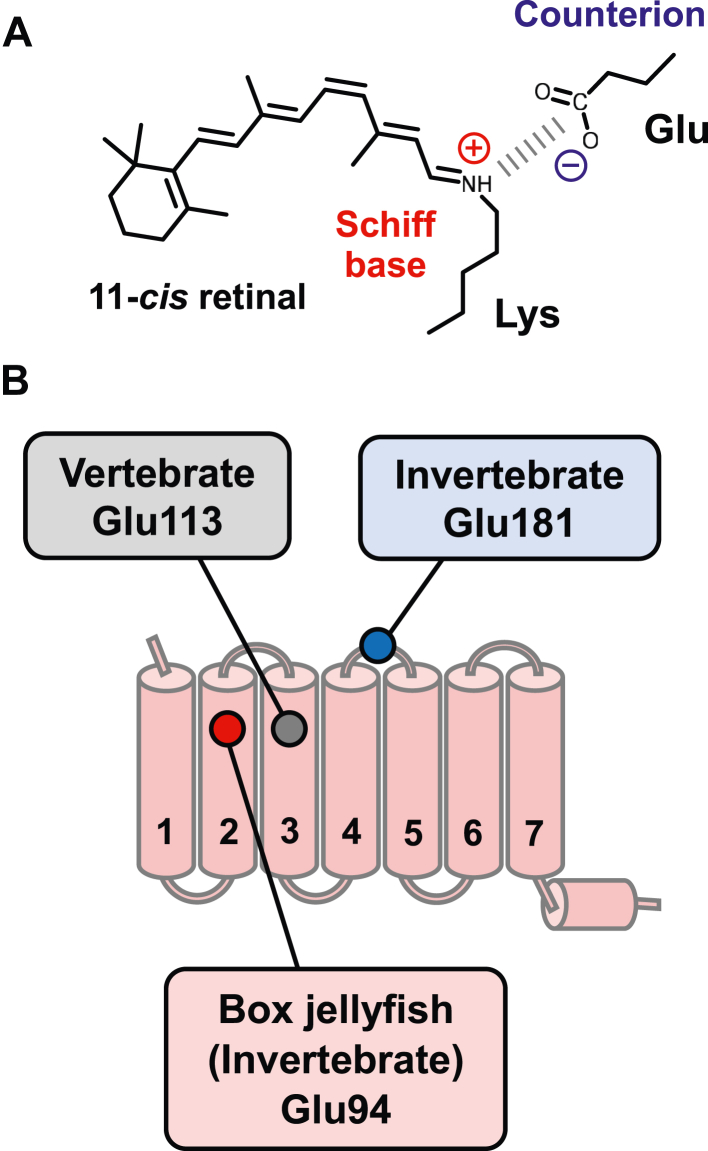
**Electrostatic interaction between retinal chromophore and counterion, and counterion displacement in animal rhodopsins.***A*, the 11-*cis*-retinal chromophore and counterion. Both vertebrate and invertebrate rhodopsins have a negatively charged glutamate near the protonated retinal Schiff base for stabilization of the positive charge. *B*, a schematic illustration of counterion displacement between vertebrate and invertebrate rhodopsins, including JelRh. The counterion in vertebrate rhodopsin is positioned at 113 in TM3, while the position 181 in the second extracellular loop corresponds to the counterion for invertebrate rhodopsin. However, the counterion in JelRh has moved to a unique position at 94 in TM2. JelRh, box jellyfish rhodopsin; TM2, transmembrane 2; TM3, transmembrane 3.

The amino acid residue Glu113 is highly conserved in vertebrate rhodopsins, while Glu181 is present in all animal rhodopsins, including those of vertebrates (with the exception of some cone opsins). This suggests that Glu181 may have been the original counterion of ancestral rhodopsins, but this is yet to be visualized structurally. As seen in the structures of squid rhodopsin (SquRh) (16) and jumping spider rhodopsin1 (SpiRh1) (17), Glu181 is located ∼6 Å from the Schiff base. Therefore, experimental and theoretical studies are still actively being pursued to elucidate the molecular mechanism by which Glu181 acts as a counterion (15).

The displacement of the counterion also affects the efficiency of G protein activation (13). Thus, this displacement from Glu181 to Glu113 results in the acquisition of a highly efficient G protein activation, which in turn increases the photosensitivity of vertebrate rhodopsins compared to invertebrate rhodopsins. It has also been found that the difference in G protein activity between monostable vertebrate rhodopsin and bistable invertebrate rhodopsin is due to the larger opening motion of TM6 on the cytoplasmic side in vertebrate rhodopsin as compared to invertebrate rhodopsin (18).

Recent research has revealed that box jellyfish, an invertebrate, having opsin which binds to retinal chromophore to form pigment called jellyfish rhodopsin (JelRh) utilizes a Glu94 (TM2), as a counterion instead of 113 (TM3, vertebrate rhodopsin) or 181 (ECL2, invertebrate rhodopsin) (19). The counterion of JelRh is positioned at the transmembrane, similar to that of vertebrate rhodopsin, although each counterion is located at different TM helices, suggesting that both of them evolved in a convergent manner. Additionally, JelRh is the first animal rhodopsin known to trigger a G_s_ protein–mediated phototransduction cascade (19, 20). Its ability to control the G_s_ signaling pathway with light makes it a promising optogenetics tool for regulating repeatedly cAMP induction in cultured HEK cells (*in vitro*) (21), fibroblast (circadian rhythms) (22), cardiomyocytes and/or whole heart (cardiac function) (23), and *C. elegans* (behavioral control *in vivo*) (24). However, the molecular mechanism behind how JelRh's photoreaction properties are affected by its unique counterion positioning and whether it is related to its ability to activate G_s_ proteins is still unknown.

In this study, we conducted low-temperature UV–visible and FTIR spectroscopic measurements on JelRh to gain structural insight into the retinal binding pocket. The main goal was to analyze the structure of the initial intermediate, Batho, formed by photoisomerization of the retinal and to compare the similarities and differences in the retinal structure, isomerization process, and protein interaction caused by the displacement of a counterion to TM2 when compared to both vertebrate and invertebrate rhodopsins. For comparison, we used bovine rhodopsin (BovRh) as a representative of vertebrate rhodopsins, and SquRh as a representative of invertebrate rhodopsins, and analyzed the structural changes induced by photoisomerization from 11-*cis* to all-*trans* and from the analog isomeric 9-*cis* to all-*trans* (Fig. 2*A*).

**Figure 2:**
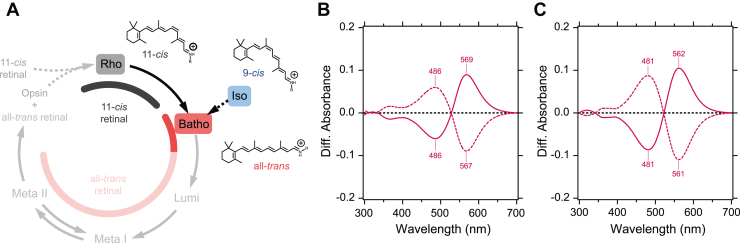
**Schematic photoreaction scheme of monostable vertebrate rhodopsin and light-induced UV-visible difference spectra of box jellyfish rhodopsin.***A*, the general photoreaction scheme of monostable vertebrate rhodopsin involves the isomerization of either the 11-*cis* or 9-*cis* bound resting states to the all-*trans* bound primary intermediate Batho state upon light illumination, followed by a photobleaching reaction through several photointermediate states, including the active Meta-II state. Light illumination at 77 K allows for the cryo-trapping of the Batho intermediate state. *B*, light-induced UV–visible difference spectra upon illumination with 500 nm light (*deep red solid line*) and with light >610 nm (*dotted line*) exhibit the photochromism between the 11-*cis* bound resting and all-*trans* bound Batho intermediate states. *C*, light-induced UV–visible difference spectra upon illumination with 500 nm light (*deep red solid line*) and with light >520 nm (*dotted line*) exhibit the photochromism between the 9-*cis* bound resting and all-*trans* bound Batho intermediate states.

## Results

### JelRh exhibits a similar photoreaction as BovRh at 77 K

The UV–visible absorption spectrum of lipid-reconstituted JelRh at 77 K (Fig. S1*A*) has an absorption maximum (λ_max_) of 500 nm, consistent with previous spectra of the solubilized condition at 277 K (20). Illumination with a 500 nm interference filter resulted in a long wavelength-shifted spectrum (Fig. S1*A*) at 514 nm, thought to be the Batho intermediate and typically observed in animal visual rhodopsins. Subsequent illumination with >610 nm light confirmed the return to the resting state (Fig. S1*A*), indicating photochromism similar to that of BovRh (25) and SquRh (26, 27). On the other hand, illumination of the Batho intermediate with >520 nm light produced a short wavelength-shifted spectrum with a λ_max_ at 493 nm, confirming the formation of the 9-*cis* bound form (so-called Iso-rhodopsin or Iso) (Fig. S1*B*). In addition, photochromism was also found between Iso-JelRh and the Batho intermediate. Figure 2*B* shows the difference spectra between the Batho intermediate and JelRh and between the Batho intermediate and Iso-JelRh, respectively. The dotted lines correspond to the respective revert photoreactions. These results indicate that JelRh exhibits a similar photoreaction as BovRh and SquRh at 77 K. It is important to note that the full photochromic characteristics of the three retinal isomers (11-*cis*, all-*trans*, and 9-*cis*) should be determined by calculating the intensity ratio of their corresponding vibrational bands, as described below, through FTIR spectroscopy.

### JelRh exhibits similar photoisomerization as BovRh at 77 K

In order to gain structural information on the retinal and its binding pocket of JelRh, as well as more detailed information on the isomerization process, we conducted light-induced FTIR difference spectroscopy at 77 K using the same light illumination conditions as in Figures 2 and S1. Details of the light illumination dependence of JelRh are described in Supporting information and Figs. S2–S4.

**Figure 3:**
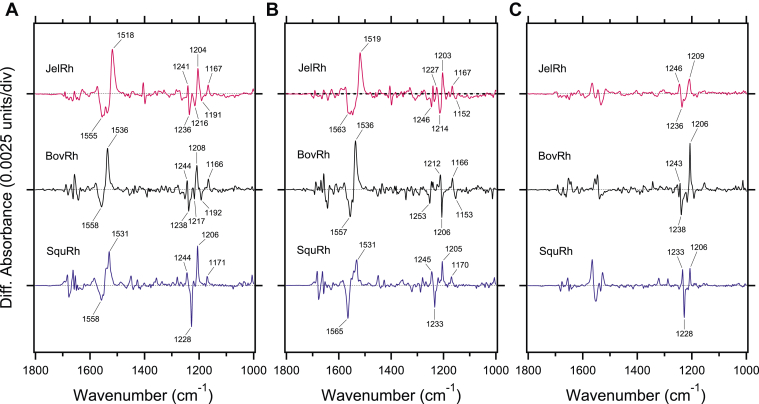
**Comparison of light-induced FTIR difference spectra of box jellyfish, bovine, and squid rhodopsins in the fingerprint vibrational mode regions.***A*, light-induced FTIR difference spectra between 11-*cis* and all-*trans* bound forms for JelRh (*top*), BovRh (*middle*), and SquRh (*bottom*) were measured at 77 K in H_2_O. These spectra indicate that positive and negative bands correspond to the Batho intermediate and 11-*cis* bound resting states, respectively. *B*, light-induced FTIR difference spectra between 9-*cis* and all-*trans* bound forms for JelRh (*top*), BovRh (*middle*), and SquRh (*bottom*) were measured at 77 K in H_2_O. These spectra indicate that positive and negative bands reflect the vibrational changes for Batho intermediate and 9-*cis* bound resting states, respectively. *C*, light-induced FTIR difference spectra between 9-*cis* and 11-*cis* bound forms for JelRh (*top*), BovRh (*middle*), and SquRh (*bottom*) were obtained by subtracting (*B*) from (*A*). The tagged bands mainly originated from retinal vibrations. Spectra of BovRh and SquRh are taken from Ref 12 and 28, respectively. One division of the y-axis corresponds to 0.0025 absorption units. The obtained spectra of JelRh, BovRh, and SquRh are normalized by 1.0, 0.1, and 1.0, respectively. BovRh, bovine rhodopsin; JelRh, box jellyfish rhodopsin; SquRh, squid rhodopsin.

Figure 3 presents the difference spectra of all-*trans* minus 11-*cis* (a) and all-*trans* minus 9-*cis* (b) for JelRh, BovRh, and SquRh. The negative bands in the difference spectra correspond to vibrations in JelRh (a) or Iso-JelRh (b) and the positive bands correspond to the Batho state. C=C stretching vibrations at 1555 (−) and 1518 (+) cm^−1^ are believed to correspond to the resting and light-illuminated Batho states of JelRh, respectively. The negative doublet peak feature is likely obscured by the amide-II band. The fingerprint region at 1500–1100 cm^−1^ exhibits bands that are mostly due to C-C stretching vibrations coupled with C-C-H bends and is highly sensitive to the isomeric state of the retinal. It is worth noting that the spectral shape of Batho/JelRh around 1200 cm^−1^ is very similar to that of Batho/BovRh but not to Batho/SquRh. The 1241 (+), 1236 (−), 1216 (−), 1204 (+), 1191 (−), and 1167 (+) cm^−1^ bands in JelRh correspond to 1244 (+), 1238 (−), 1217 (−), 1208 (+), 1192 (−), and 1166 (+) cm^−1^ bands in BovRh, which have been attributed by Raman and IR spectroscopy as shown in Table 1. These results indicate that the isomerization from 11-*cis* to all-*trans* retinal in JelRh is similar to that of BovRh.

**Table 1:** Vibrational band estimation for the FTIR difference spectra of 11-*cis* bound (resting), all-*trans* bound (Batho), and 9-*cis* bound (resting) states of JelRh

JelRh (predicted)		
Product	Frequency (cm^−1^)	Assignment
11-*cis* (Resting)	954	HC_11_=C_12_H wag
	972	
	1191	C14-C15
	1216	C8-C9
	1236	15-H
all-*trans* (Batho)	856	nd
	903	11-H wag
	910	11-H + 14-H wag
	935	HC_11_=C_12_H wag
	1167	C10-C11
	1204	C14-C15
	1241	nd
9-*cis* (Resting)	952	HC_7_=C_8_H + 12-H wag
	963	HC_7_=C_8_H wag
	1152	nd
	1214	C14-C15
	1246	nd

Each vibrational band estimation was referred from the previous FTIR spectroscopy (67, 68).

On the other hand, the spectral feature of Batho/Iso-JelRh differs from that of Batho/Iso-BovRh and Batho/Iso-SquRh. The 1246 (−), 1214 (−), and 1152 (−) cm^−1^ bands are thought to correspond to 1253 (−), 1206 (−), and 1153 (−) cm^−1^ in Iso-BovRh, respectively. However, as there is a possibility that other bands are obscured in this frequency region, double difference spectra of the 11-*cis* and 9-*cis* forms were calculated for clearer comparison and the results are shown in Figures 3*C* and S5. In Figs. S3*C* and S5, positive and negative bands correspond to the 9-*cis* and 11-*cis* forms, respectively. As clearly seen, the spectral feature of JelRh is similar to BovRh, but not to SquRh. The 1246 (+) and 1210 (+) cm^−1^ bands originated from Iso-JelRh correspond to 1243 (+) and 1206 (+) cm^−1^ bands in Iso-BovRh, respectively. Therefore, these results suggest that the manner of isomerization from 9-*cis* to all-*trans* retinal in JelRh is also similar to that of BovRh.

**Figure 4:**
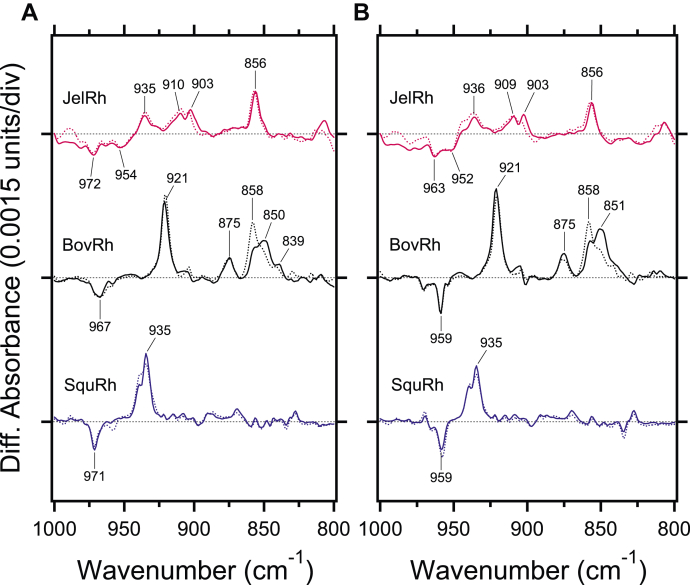
**Comparison of light-induced FTIR difference spectra of box jellyfish, bovine, and squid rhodopsins in the hydrogen-out-of-plane vibrational mode regions.***A*, light-induced FTIR difference spectra between 11-*cis* and all-*trans* bound forms for JelRh (*top*), BovRh (*middle*), and SquRh (*bottom*) in the 1000–800 cm^−1^ region measured at 77 K in H_2_O (*solid lines*) and D_2_O hydration (*dotted lines*), respectively. *B*, light-induced FTIR difference spectra between 9-*cis* and all-*trans* bound forms for JelRh (*top*), BovRh (*middle*), and SquRh (*bottom*) in the 1000–800 cm^−1^ region measured at 77 K in H_2_O (*solid lines*) and D_2_O (*dotted lines*), respectively. Each spectrum was taken from Figure 4. One division of the y-axis corresponds to 0.0015 absorption units. BovRh, bovine rhodopsin; JelRh, box jellyfish rhodopsin; SquRh, squid rhodopsin.

The torsional structure of the retinal chromophore is reflected in 1000–800 cm^−1^ region where the HOOP vibration appears. Notably, the spectral feature of the HOOP vibrations in JelRh is similar to BovRh but not to SquRh as shown in Figure 4 and Table 2. At least this phenomenon may correspond to the fact that JelRh does not exhibit bistability (20). In general, the HOOP vibrations intensify upon retinal geometric distortion, and the increase in the number of HOOP vibrations corresponds to multiple distortions along the polyene chain of the retinal chromophore. The difference spectra of Batho-minus-JelRh (Fig. 4*A*) and Batho-minus-Iso-JelRh (Fig. 4*B*) showed two negative and four positive HOOP vibrations, similar to BovRh. As shown in Table 1, the negative 972 cm^−1^ and 963 cm^−1^ bands are HOOP vibrations characteristic of the 11-*cis* and 9-*cis* isomers, respectively. On the other hand, the band intensity was reduced, and the observed band frequencies were different from those of BovRh. This suggests that the way the distortion of the retinal structure differs between JelRh and BovRh, although both rhodopsins share the fact that the retinal structure is distorted. Conversely, SquRh exhibits bands at 971 and 935 cm^−1^ on the negative and positive sides, respectively, corresponding to BovRh's 967 and 921 cm^−1^, but no other HOOP bands are detected. This indicates that the retinal chromophore has an undistorted structure, which is a hallmark of bistable rhodopsins that undergo reversible photoreaction upon exposure to light (28, 29). Thus, our results suggest that JelRh has a monostable vertebrate visual rhodopsin-like retinal structure as well as the mechanism of retinal isomerization.

**Table 2:** The calculated total area of both negative and positive HOOP vibrational bands appeared in the 1000–800 cm^−1^ region in each rhodopsin

Area of HOOP band appearing in the 1000–800 cm^−1^		
Animal rhodopsins	S (11-*cis* and all-*trans*)	S (9-*cis* and all-*trans*)
JelRh	0.01949	0.01863
BovRh	0.01991	0.02371
SquRh	0.01472	0.01283

**Figure 5:**
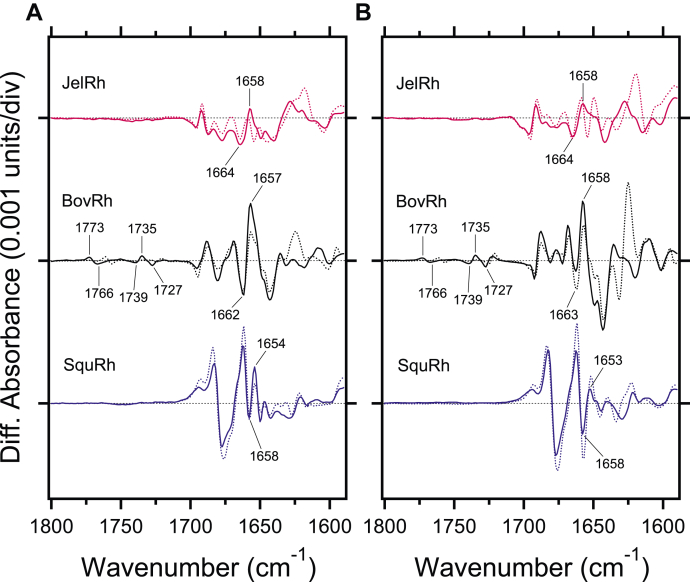
**Comparison of light-induced FTIR difference spectra of box jellyfish, bovine, and squid rhodopsins in the carboxylic acid side-chain vibrational mode regions.***A*, light-induced FTIR difference spectra between 11-*cis* and all-*trans* bound forms for JelRh (*top*), BovRh (*middle*), and SquRh (*bottom*) in the 1800–1600 cm^−1^ region measured at 77 K in H_2_O (*solid lines*) and D_2_O hydration (*dotted lines*), respectively. *B*, light-induced FTIR difference spectra between 9-*cis* and all-*trans* bound forms for JelRh (*top*), BovRh (*middle*), and SquRh (*bottom*) in the 1800–1600 cm^−1^ region measured at 77 K in H_2_O (*solid lines*) and D_2_O (*dotted lines*), respectively. Each spectrum was taken from Figure 4. One division of the y-axis corresponds to 0.001 absorption units. BovRh, bovine rhodopsin; JelRh, box jellyfish rhodopsin; SquRh, squid rhodopsin.

The C=N stretching vibrations of the protonated retinal Schiff base are observed in 1650–1600 cm^−1^ region (Fig. 5). These vibrations are sensitive to hydrogen (H)/deuterium (D) exchange, and the difference in frequency is considered as a probe for the strength of the hydrogen bond (30, 31); the larger the difference, the stronger the bond. In BovRh, previous low-temperature resonance Raman spectroscopy determined the vibrational frequencies of the C=NH and C=ND stretches at 1656 cm^−1^ and 1623 cm^−1^, respectively, with a difference of 33 cm^−1^. Upon the formation of the Batho state in BovRh, these vibrations are observed at 1654 cm^−1^ and 1626 cm^−1^, with a difference of 28 cm^−1^. This indicates that the hydrogen bonding strength of the protonated Schiff base is almost the same in the resting and Batho states (32). Therefore, in the FTIR difference spectra between the resting and Batho states, it is difficult to accurately observe the vibrational band of the Schiff base. Additionally, the C=O stretching vibrations of the α-helix main chain, referred to as amide-I bands, which reflect changes in the protein backbone, will also overlap in the same frequency region. Similarly, the C=NH stretching vibration could not be identified in both JelRh and SquRh (Fig. 5). As a result, the hydrogen bond strength of the Schiff base could not be derived. However, it is worth noting that the N-D stretching vibration of the Schiff base in D_2_O provides more direct information about the hydrogen bond of the Schiff base, as described below.

X-ray crystallography (33), NMR (34, 35), and various spectroscopic analyses (36) of BovRh have shown that the conformational changes in the protein moiety associated with the formation of the Batho intermediate are limited to localized areas around the retinal binding pocket. Nevertheless, differences in amide-I bands have been observed among the three-cone opsins, blue, green, and red (37, 38), which are thought to be due to differences in the structure of the retinal binding pocket and/or retinal–protein interaction. Therefore, we can expect to verify differences in the local structure of the retinal binding pocket by using changes in the amide-I band as a probe. From Figure 5*A*, the amide-I band of JelRh is considered to be 1664 (−)/1658 (+) cm^−1^, and this paired band is similar to BovRh (1662 (−)/1657 (+) cm^−1^) and SquRh (1658 (−)/1654 (+) cm^−1^). In addition, this frequency is characteristic of an α_II_ helix (39), which is also common to the cone opsin of animal rhodopsin (37, 38). It should be noted that the spectral changes of amide-I vibrations at <1650 cm^−1^ are much smaller in both JelRh and SquRh than in BovRh. This suggests that the local structural changes of the peptide backbone in JelRh and SquRh upon retinal isomerization are very small.

Hydrogen bonding changes and protonation reactions of carboxylic acids can be also monitored in the >1700 cm^−1^ region, where protonated carboxylic acid C=O stretching vibrations are observed. For BovRh, the pair bands at 1773 (+)/1766 (−) cm^−1^ and 1739 (−)/1735 (+)/1727 (−) cm^−1^ are attributed to the C=O stretching vibrations of Asp83 in TM2 and Glu122 in TM3, respectively (40). These two carboxylic acids are well known to organize the key hydrogen bonding network between the extracellular retinal binding pocket and cytoplasmic G protein–interacting site (41, 42, 43). Interestingly, in JelRh and SquRh, no bands are observed in the 1800–1700 cm^−1^ region that originates from hydrogen bond changes in protonated carboxylic acids. At least from the amino acid sequence (Fig. S6), it makes sense that Asp at position 83 is conserved for JelRh and SquRh, but Glu at position 122 is replaced by Gln and Phe, respectively, so the Glu122 band is not observed for both cases. On the other hand, the reason for the observed lack of hydrogen bond changes in Asp83 in JelRh and SquRh suggests that either no conformational change occurs near position 83 or Asp83 is deprotonated during the early photoreaction step associated with Batho state formation.

### JelRh exhibits a similar hydrogen bonding network at the retinal binding pocket as BovRh

**Figure 6:**
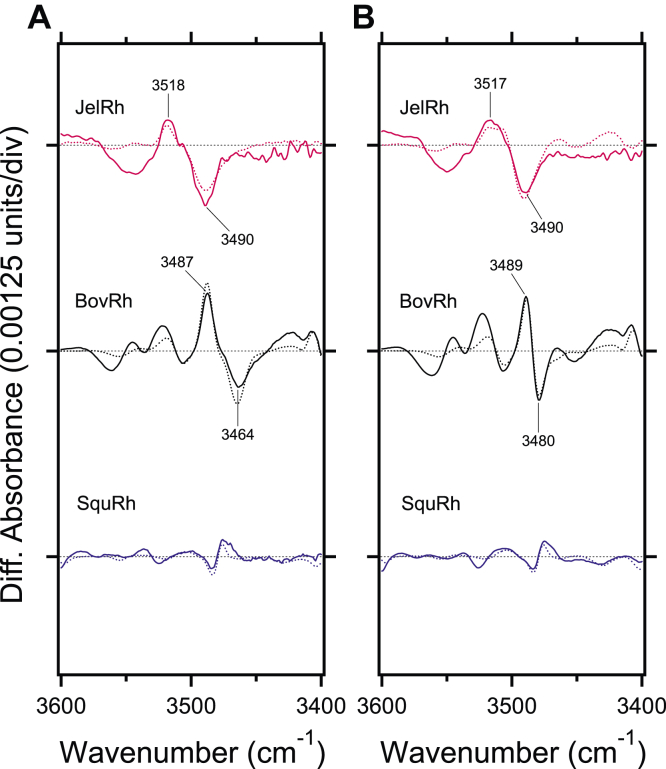
**Comparison of light-induced FTIR difference spectra of box jellyfish, bovine, and squid rhodopsins in the hydrophilic amino acid side-chain vibrational mode regions.***A*, light-induced FTIR difference spectra between 11-*cis* and all-*trans* bound forms for JelRh (*top*), BovRh (*middle*), and SquRh (*bottom*) in the 3600–3400 cm^−1^ region measured at 77 K in H_2_O (*solid lines*) and D_2_O hydration (*dotted lines*), respectively . *B*, light-induced FTIR difference spectra between 9-*cis* and all-*trans* bound forms for JelRh (*top*), BovRh (*middle*), and SquRh (*bottom*) in the 3600–3400 cm^−1^ region measured at 77 K in H_2_O (*solid lines*) and D_2_O (*dotted lines*), respectively. Each spectrum was taken from Figure 4. One division of the y-axis corresponds to 0.00125 absorption units. BovRh, bovine rhodopsin; JelRh, box jellyfish rhodopsin; SquRh, squid rhodopsin.

Observations of changes in the amino acid side chains that constitute the retinal binding pocket can also provide insight into the effects of counterion displacement. Figure 6 shows the X-H stretching vibration region where O-H and N-H groups of hydrophilic amino acids are observed. The pair bands at 3487 (+)/3464 (−) cm^−1^ (Figs. 6*A*) and 3489 (+)/3480 (−) cm^−1^ (Fig. 6*B*) in BovRh and Iso-BovRh are attributed to the O-H stretching vibration of Thr118, which is a D_2_O unexchangeable band (44). A frequency shift of 16 cm^−1^ is observed at negative 3464 cm^−1^ and 3480 cm^−1^, indicating a difference in the hydrogen bonding strength of Thr118 between BovRh and Iso-BovRh. In fact, the OH group of Thr118 forms a hydrogen bond with the backbone carbonyl oxygen of Gly114 at a distance of 2.8 Å in the 11-*cis* bound form (PDB: 1U19 (11)), and 2.9 Å in the 9-*cis* bound form (PDB: 2PED (45)), indicating differences in hydrogen bond strength. Notably, SquRh has no significant bands observed in the 3600–3400 cm^−1^ region. The amino acid at position 118 in SquRh has been replaced by Gly, which is also consistent with the missing O-H stretching vibration band corresponding to this frequency region. In the present study, Figure 6 shows that pair bands of 3518 (+)/3490 (−) cm^−1^ and 3517 (+)/3490 (−) cm^−1^ were observed in JelRh and Iso-JelRh, respectively. Since JelRh has threonine at position 118 like BovRh, these paired bands can be attributed to the O-H stretching vibration band of Thr118. The hydrogen bonding partner of Thr118 could be the C=O oxygen atom of the main chain of Gly114 as in BovRh. The up-shift of the vibrational frequency of Thr118 O-H stretch in JelRh compared to BovRh suggests that the hydrogen bonding strength between Gly114 and Thr118 is weaker, that is, the distance between the oxygen atom of the main chain of Gly114 and the hydrogen atom of the side chain of Thr118 is longer than that of BovRh.

Interestingly, in contrast to BovRh, JelRh shows that the O-H stretching vibration bands originating from Thr118 in the 11-*cis* (Fig. 6*A*) and 9-*cis* (Fig. 6*B*) forms have identical frequencies. This indicates that the chemical differences in retinal isomers do not affect the interaction between Gly114 and Thr118. Given that position 118 is at a distance that could potentially interact with the C_9_ of the retinal, in JelRh, it is assumed that the spatial distance between the retinal polyene chain and Thr118 is longer, suggesting that the space around the polyene chain is large. This result is consistent with the fact that the amide-I band and HOOP vibration of JelRh have smaller intensities than those of BovRh. Additionally, since the O-H stretching vibration bands of Thr118 in JelRh is D_2_O unexchangeable, similar to BovRh, this suggests that the protein environment surrounding Thr118 is very hydrophobic.

**Figure 7:**
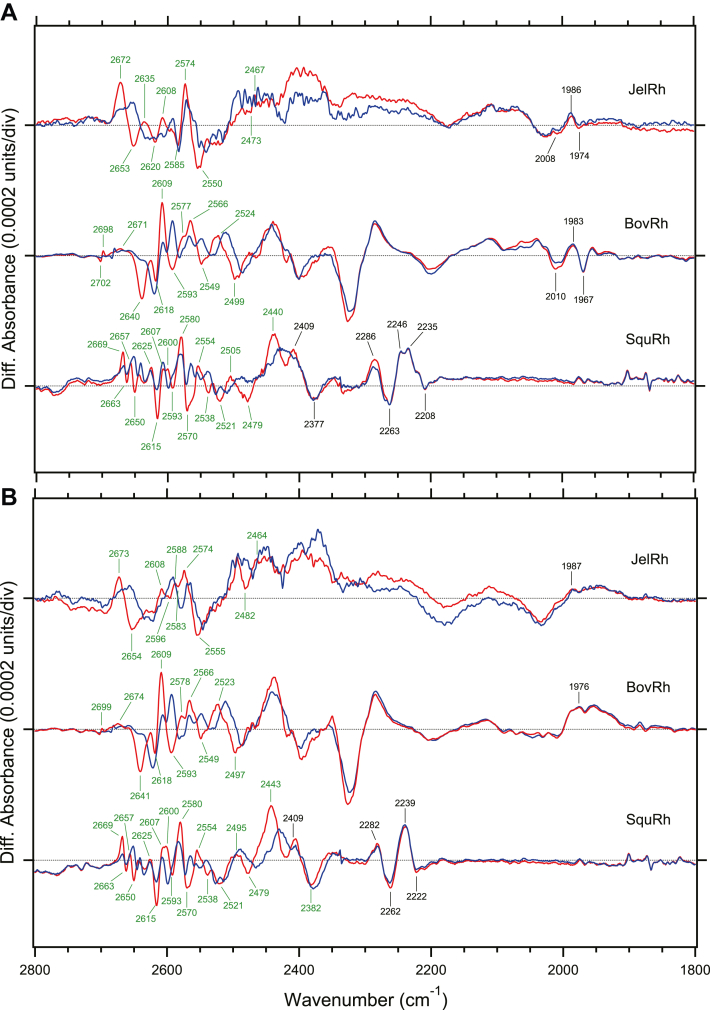
**Comparison of light-induced FTIR difference spectra of box jellyfish, bovine, and squid rhodopsins in the protein-bound water vibrational mode regions.***A*, light-induced FTIR difference spectra between 11-*cis* and all-*trans* bound forms for JelRh (*top*), BovRh (*middle*), and SquRh (*bottom*) in the 2800–1800 cm^−1^ region measured in D_2_O (*red lines*) and D_2_^18^O hydration (*blue lines*), respectively. *B*, light-induced FTIR difference spectra between 9-*cis* and all-*trans* bound forms for JelRh (*top*), BovRh (*middle*), and SquRh (*bottom*) in the 2800–1800 cm^−1^ region measured in D_2_O (*red lines*) and D_2_^18^O (*blue lines*), respectively. One division of the y-axis corresponds to 0.0002 absorption units. The *green-tagged bands* exhibit the isotope effect by ^18^O and are attributed to the O-D stretching vibration of protein-bound water. BovRh, bovine rhodopsin; JelRh, box jellyfish rhodopsin; SquRh, squid rhodopsin.

In BovRh, internal water molecules are important components of the retinal binding pocket. They play a role in the stabilization of the salt bridge between protonated Schiff base and the counterion (11, 12), and in the formation of a hydrogen bonding network with key amino acids (Asp83 and Glu122) that are involved in BovRh activation (11, 40). The crystal structure of SquRh and/or SpiRh1 also show that a chain of water molecules extends through Asp83 to fill the interhelical cavity between the retinal and Asn302 that constitutes the NPxxY motif and is important for the G protein activity (16, 17). This indicates that water molecules play a role in linking the conformational changes in the retinal binding site to the changes in the cytoplasmic side that interacts with the G protein. Notably, these protein-bound water molecules observed in the crystal structure of BovRh and SquRh are also detected as water O-D stretching vibrations by FTIR spectroscopy (Fig. 7, (12, 46)). The number of water molecules that are affected by the formation of the Batho intermediate in close proximity to the retinal chromophore is higher in SquRh compared to BovRh, as indicated by the differences in the water O-D stretching vibrations. In particular, BovRh exhibits six vibrations on the negative side and six on the positive side, while SquRh displays eight and nine, respectively. This result aligns with the observation of a chain of water molecules *via* Asp83, which was seen only in SquRh in the crystal structure (16).

As depicted in Figure 7, we were able to identify the water O-D stretching vibrational bands in JelRh. The bands that shift due to ^18^O isotope labeling of water (depicted by the blue line) have been assigned as the O-D stretching vibrational bands of water molecules. In the Batho-minus-JelRh difference spectra, there are five O-D stretching vibrations of water at negative 2653, 2620, 2585, 2550, and 2473 cm^−1^ and positive 2672, 2635, 2608, 2574, and 2467 cm^−1^. The Batho-minus-Iso-JelRh difference spectra show five water O-D stretching bands at 2654, 2596, 2583, 2555, and 2482 cm^−1^ on the negative side and five water O-D stretching bands at 2673, 2608, 2588, 2574, and 2464 cm^−1^ on the positive side. In BovRh, the negative 2618 (−) and 2640 (−) cm^−1^ bands are believed to originate from water molecules located near Glu113 and Asp83/Gly120, respectively (12, 40). In JelRh, the corresponding water bands are thought to be 2620 (−) and 2653 (−) cm^−1^ bands, respectively. Additionally, similar to BovRh, no O-D stretching vibration bands of water were observed in the 2300 cm^−1^ > region. Since the observed water bands are located at the high-frequency region (2700–2450 cm^−1^), they correspond to weakly hydrogen-bonded water molecules. This indicates that there are no water molecules that form direct hydrogen bonds with the retinal Schiff base, as observed in microbial rhodopsin (47). In other words, the direct counterpart of the retinal Schiff base of JelRh is likely to be the counterion Glu94. The O-D stretching vibrational bands of water is not observed in the isomer difference, as shown in the 9-*cis* -minus- 11-*cis* bound form spectra (Fig. S7). This behavior is similar to BovRh, suggesting that there is no protein-bound water present near the polyene chain of the retinal chromophore in JelRh. In contrast, for SquRh, a water-originating signal was observed in the double difference spectra between 11-*cis* and 9-*cis* forms at 2451 and 2389 cm^−1^, respectively (Fig. S7 (46)). This indicates the presence of water molecules near the retinal chromophore in SquRh. The observed vibrational frequencies indicate that water molecules form strong hydrogen bonds, and it has been proposed previously that water molecules exist in positions where they can form direct hydrogen bonds with the retinal Schiff base (30).

In Figure 7, the vibrational bands observed in the 2200 cm^−1^ region (black tags) are believed to be the N-D stretching vibrational bands of the protonated retinal Schiff base. This is supported by previous assignments of the N-D stretching vibration of primate green cone opsin through C_15_-D isotope labeling of retinal (2099 cm^−1^ band) (48). In JelRh, a band is observed at 2008 and 1974 cm^−1^, which is similar to that of BovRh at 2010 and 1967 cm^−1^, indicating that the interaction strength between the Schiff base and counterion is comparable in both, despite the difference in counterion position. The N-D stretching vibration band in the Batho state of JelRh is thought to be 1986 cm^−1^, similar to 1983 cm^−1^ in BovRh, showing little change in interaction strength between the Schiff base and counterion in both states. However, in SquRh, the N-D stretching vibration band is observed around 2200 cm^−1^, indicating a weaker interaction between the Schiff base and counterion, likely due to the counterion Glu181 being located 6.7 Å away from the Schiff base, as observed in the crystal structure of SquRh (16).

## Discussion

Counterions in rhodopsin play a vital role in not only stabilizing the protonated Schiff base of retinal but also in exerting functions such as spectral tuning, isomerization control, and activation control. In this study, we present structural information on the retinal binding pocket of JelRh, which has a counterion in TM2. We examined the isomerization of the retinal and the conformational changes induced within the retinal binding pocket and the protein moiety. By comparing JelRh to BovRh and SquRh, we aim to clarify whether JelRh exhibits similar spectral properties to those of vertebrate rhodopsin or invertebrate rhodopsin.

**Figure 8:**
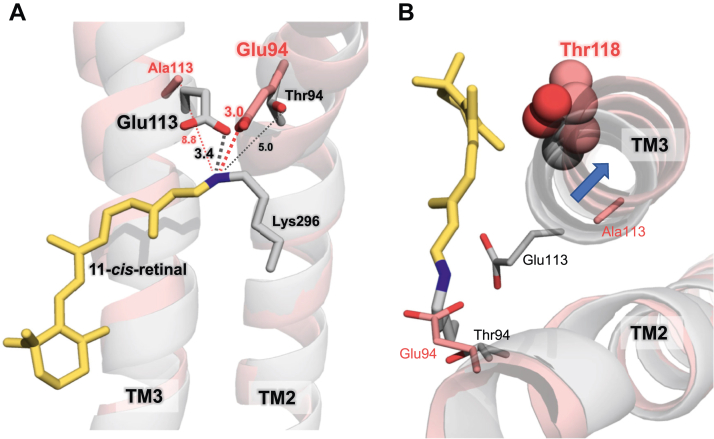
**The detailed comparison of the Schiff base region between the predicted model of jellyfish rhodopsin and X-ray structure of bovine rhodopsin.***A*, structural comparison between AlphaFold2 3D model of JelRh (*pale pink*) and BovRh (PDBID: 1U19 (11); *light gray*), viewed from the helix 5/6 side. The *upper* and *lower* regions depict the extracellular and cytoplasmic sides, respectively. The 11-*cis*-retinal chromophore is from BovRh (PDBID:1U19 (11)), as AlphaFold2 cannot reconstruct a model structure including 11-*cis*-retinal. The amino acids shown correspond to the counterions of JelRh (Glu94 in TM2) and BovRh (Glu113 in TM3), respectively. *B*, structural comparison between AlphaFold2 3D model of JelRh (*pale pink*) and BovRh (*light gray*), viewed from the extracellular side. Thr118 in both JelRh and BovRh is illustrated by a space-filling model. The TM3 of JelRh is shifted 1.5 Å out of the membrane compared to that of BovRh, resulting in a widened retinal binding pocket in JelRh. BovRh, bovine rhodopsin; JelRh, box jellyfish rhodopsin; SquRh, squid rhodopsin.

In reference to spectral tuning, the λ_max_ of JelRh is 500 nm, which is almost identical to that of BovRh (498 nm) but not to SquRh (480–482 nm) (Fig. S1). Additionally, JelRh and BovRh with counterions in the TM region show similar λ_max_. This suggests that the strength of the interaction between the protonated Schiff base and the counterion in JelRh and BovRh is similar. In this study, we used FTIR to compare the three rhodopsins with respect to their N-D stretching vibration of protonated Schiff bases, which is an indicator of the interaction strength between the Schiff base and counterions. As expected, the frequencies of the N-D stretching vibration of JelRh and BovRh are similar (2008 and 1974 cm^−1^ for JelRh and 2010 and 1967 cm^−1^ for BovRh). Can these results be explained structurally? Since the structure of JelRh has not yet been determined, we created a 3D model of JelRh using Alpha Fold2 (AF2) (49) for the structural comparison in Figure 8. Interestingly, in the BovRh structure, Thr (TM2) at position 94, which is the counterion position in JelRh, is 5.0 Å away from the retinal Schiff base. Whereas in the AF2 3D model of JelRh, Glu at position 94 is 3.0 Å away from the retinal Schiff base, similar to the distance between the Schiff base and Glu113 in BovRh (3.4 Å). This is consistent with the result that the N-D stretching frequencies observed in the present FTIR measurements are similar for both, *i.e.*, the hydrogen bond strengths are similar. Structural comparisons also suggest that TM2 in the extracellular region of JelRh is located slightly inward toward the retinal Schiff base in comparison to BovRh, situating Glu94 in JelRh at a hydrogen bond distance to the Schiff base.

**Figure 9:**
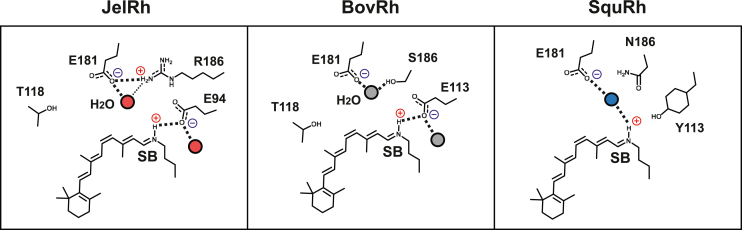
**Structural comparisons of the retinal binding pocket among each rhodopsin at 77 K are depicted from the results from the FTIR measurements.** Protein-bound water molecules are represented by colored circles. Despite the different positions of the counterions, the hydrogen bonding networks in the Schiff base region of JelRh and BovRh show a similar structural architecture. Structural similarities between JelRh and BovRh are also observed in the protein environment in the region along the retinal polyene chain and around the amino acid at position 118. BovRh, bovine rhodopsin; JelRh, box jellyfish rhodopsin; SquRh, squid rhodopsin.

The position 113 in JelRh is replaced by an alanine residue, and the methyl group of the alanine side chain is 8.8 Å away from the Schiff base in comparison to BovRh, which supports the idea that position 113 in JelRh cannot function as a counterion. Gerrard *et al.* (19) previously observed absorption in the UV (27%) and visible (73%) regions of the action spectra when measuring activity against the E94Q/A113E double mutant, suggesting the possibility of a counterion switch. However, when we expressed and purified E94Q/A113E double mutant and performed hydroxylamine bleach measurements, we only observed absorption in the UV region from the detergent-solubilized and purified sample and not in the visible region (Fig. S8, *A* and *B*). The addition of an acid to the sample resulted in a red-shift around 440 nm due to the Schiff base protonation, indicating an intact, deprotonated Schiff base (Fig. S8*C*). This suggests that the distance between the Schiff base and position 113 in JelRh destabilizes the protonated Schiff base. In fact, about 30% of the Schiff base is deprotonated and absorbs in the UV region when measured in cells as shown in Gerrard *et al.* (19) Additionally, Gerrard *et al.* also tested whether Glu181 could function as a counterion for JelRh. The activity assay for the E94Q/R186V double mutant in JelRh detected a peak in the visible region, reproducing the counterion of invertebrate rhodopsins such as SquRh and SpiRh1, indicating functional redundancy in the counterion position. However, when we attempted to express and purify the E94Q/R186S double mutant, we obtained similar results as the E94Q/A113E double mutant (Fig. S8, *A* and *B*), further supporting the idea that JelRh has evolved independently and that position 94 is indeed the optimal counterion position for JelRh. Based on our current FTIR results and the AF2 3D model structure of the JelRh, we propose a schematic architecture of the retinal binding pocket of JelRh (Fig. 9). Like BovRh, the protonated Schiff base of JelRh is stabilized by the proximal Glu94 as a counterion through direct electrostatic interaction. Although our FTIR results do not allow us to experimentally identify it, we propose that in JelRh there are also water-mediated interactions bridging Glu181 to Ser186 and the retinal Schiff base, as observed in BovRh. Additionally, since the O-D stretching vibration band originating from water molecules in the vicinity of Glu113 observed in BovRh was also observed in the spectra of JelRh, we assume the existence of a water molecule in the vicinity of Glu94 that stabilizes the Schiff base region.

The regulation of retinal isomerization is examined by comparing FTIR difference spectra of JelRh and BovRh. The results show that both proteins exhibit similar C-C stretching vibration and HOOP band features, although there are differences in signal intensity and vibrational frequency. This indicates that JelRh and BovRh have similar retinal isomerization. The distorted structure of the retinal chromophore plays a crucial role in achieving ultra-fast "*cis*-*trans*" isomerization (50, 51) and one of the factors that cause this distortion is the strength of the interaction between the Schiff base and the counterion (52). The HOOP band reflects the distorted structure of the retinal and the similarity of the HOOP bands between JelRh and BovRh strongly suggests that their retinal distorted structures are similar. This result is consistent with the conclusion in spectral tuning that the strength of the interaction between the Schiff base and counterion is comparable in both proteins.

In addition to the counterion, amino acids that make up the retinal binding pocket also play an important role in the isomerization of the retinal. However, despite the similarity in retinal isomerization between JelRh and BovRh, this study found that the structural changes in the retinal binding pocket induced by retinal isomerization were different. The frequency of the amide-I band of JelRh is similar to that of BovRh and SquRh, but the intensity of the amide-I band is about one-third of BovRh. This suggests that the conformational change upon *cis-trans* isomerization is smaller. In other words, the retinal binding pocket in JelRh is wider than BovRh. This result aligns with the observation that the O-H stretching vibration of Thr118, a component of the retinal binding pocket, has the same frequency in both the 11-*cis* and 9-*cis* forms in JelRh. Additionally, the AF2 3D model of JelRh reveals that TM3 is located farther away from the retinal polyene chain in comparison to BovRh, causing Thr118 to also shift away from the retinal polyene chain, resulting in an expanded retinal binding pocket (Fig. 8*B*). This is further supported by the FTIR results.

The reorganization of the hydrogen-bonding network formed by central water molecules within the transmembrane region plays an important role in the regulation of rhodopsin activation Asp83, located in TM2 and highly conserved in class A GPCRs, is linked *via* a water molecule to Asn302, an NPxxY motif important for GPCR activation. Interestingly, a protonated C=O stretching vibration corresponding to the hydrogen bond change of Asp83 is observed on the difference spectra of the Batho intermediate in BovRh (Fig. 5). This result indicates that the conformational changes triggered by retinal isomerization on the extracellular side are already transmitted to Asp83 during the formation of the Batho intermediate. However, no Asp83-derived vibrational bands were observed on the JelRh and SquRh spectra. Since Asp83 is located inside the transmembrane helix, it is unlikely that it would be deprotonated. Some class A GPCRs, such as A_2_AR (53), β_1_AR (54), δOR (55), and PAR1 (56), are known to regulate GPCR activity by binding of Na ions (Na^+^) near Asp83. In this case, Asp83 is considered to be deprotonated by the binding of Na^+^, but it is still considered to be protonated in the Na^+^-free state.

The absence of vibrational changes in Asp83 as observed in both JelRh and SquRh may be due to either (1) the isomerization changes in the retinal chromophore during the formation of the Batho intermediate not being transmitted to Asp83 or (2) that the hydrogen bonding changes around Asp83, crucial for activation in class A GPCRs like BovRh, have already occurred in the resting state, turning it into a pre-active state. Regarding the former possibility, it is challenging to believe that the change in retinal isomerization is not transmitted, as the hydrogen bonding network through water molecules extends from the retinal binding pocket to Asp83 in the crystal structure of SquRh (16) or SpiRh1 (17). In contrast, the crystal structure of SquRh (16) shows that Tyr223 in TM5 and the NPxxY motif Tyr306 in TM7 are oriented towards the transmembrane core domain, similar to the active conformation of BovRh and GPCRs (Fig. S9). Additionally, similar changes were also observed for Asp83 in Fig. S9. These structural features are also confirmed in the recently determined SpiRh1 (17). It is also noteworthy that the AF2 3D model structure of JelRh has Tyr306 and Asp83 oriented similarly to the active conformation of BovRh (Fig. S9), suggesting that JelRh, like SquRh and SpiRh1, has already taken on a pre-active conformation in the resting state. This is why the hydrogen bond change in Asp83 of JelRh and SquRh was not observed.

On the other hand, in the AF2 3D model of JelRh, the side chain of Tyr223 is oriented outward from the membrane, similar to the resting state of BovRh. Tyr223 plays an important role in stabilizing the Meta-II intermediate by forming a strong hydrogen bond with Arg135 of the D(E)RY motif during BovRh activation (57, 58, 59). Given that the side chain of Tyr223 is facing the transmembrane domain core in SquRh and SpiRh1, the cytoplasmic side region of TM5 in JelRh is closer to the resting state. In combination with the conformational states of TM2 and TM7 as previously described, JelRh has an intermediate conformation between BovRh and SquRh (Fig. S9). These findings from the AF2 3D model of JelRh are also consistent with the intermediate spectral features of JelRh observed in the FTIR difference spectra when compared to BovRh and SquRh.

JelRh is a unique animal rhodopsin that couples with G_s_ protein. Research has shown that the location of Phe139 at the beginning of the ICL2 helix is crucial for its interaction with G_s_ proteins (60). Additionally, studies have revealed that a mutant form of β_2_AR (F139A) has a greatly reduced ability to couple with G_s_ proteins (61). Furthermore, recent molecular dynamics simulations conducted by Sandhu *et al.* (62) have indicated that certain amino acids in the TM5 region, in addition to those in ICL2, play a role in selective binding to G_s_ proteins. In particular, Glu229 and Lys236 contribute to this binding by interacting with specific residues of the G_s_ protein (Gln384 and Asp381). A comparison of the amino acid sequences of ICL2 and TM5 in JelRh, BovRh, SquRh, and β_2_AR (Fig. S10) revealed that JelRh also has conserved residues Phe143 in ICL2, Glu229 in TM5, and Arg236. Bailes *et al.* (22) have reported that a change in Phe112 (corresponding to Phe143 in BovRh) to alanine (F112A) results in a shift toward signaling *via* β-arrestins and a reduction in G_s_ coupling. Therefore, it is likely that JelRh activates G_s_ proteins through a similar mechanism as other G_s_-coupled GPCRs such as β_2_AR. However, the retinal isomerization and the resulting conformational changes in the retinal binding pocket as observed through FTIR spectroscopy of JelRh are similar to those seen in G_t_-coupled BovRh. To fully understand the mechanism of G_s_ protein activation by JelRh, further studies are necessary, including spectral comparisons of intermediates from the Batho intermediate.

## Conclusion

Understanding the light activation mechanism of JelRh, the only animal rhodopsin with G_s_-cAMP signaling pathway, will not only improve our understanding of the activation process of G_s_-coupled GPCRs but also offer a new optogenetic tool for light-regulated cAMP induction. In this study, the first structural examination of JelRh was conducted to investigate the effect of JelRh gaining a counterion in TM2 on its function. Although JelRh gained a counterion in TM2 independent of other animal rhodopsins, we compared its light-activation mechanism with the vertebrate rhodopsin, BovRh, and the invertebrate rhodopsin, SquRh. Results indicate that the manner of retinal isomerization occurring upon photoabsorption and the retinal structure of JelRh are similar to those of BovRh, rather than SquRh. The strength of the interaction between the retinal Schiff base and the counterion is also almost identical to that of BovRh. JelRh displays a unique hydrogen bonding network, formed by amino acids and water molecules around the retinal chromophore, indicative of a hybrid behavior combining both BovRh-like and SquRh-like structural changes. Further structural studies of the active state will be performed in the future to clarify the molecular mechanism that enables G_s_ coupling.

## Experimental procedures

### Sample expression and purification

The box jellyfish *Carybdea rastonii* opsin gene (AB435549) was subcloned from a pcDNA3.1 vector to a pFastBac HT vector (Bac-to-Bac, Thermo Fischer Scientific). The monoclonal antibody rho 1D4 epitope sequence (TETSQVAPA from BovRh) gene (63) was linked at C terminal region to aid in protein purification. Following the manufacturer's instructions, the modified gene was introduced into DH10Bac-competent cells to create Bacmid DNA, which was then transfected into Sf9 insect cells using FuGENE (Promega). After 3 days, the supernatant was collected and the recombinant baculovirus was stored at 4 °C. The Sf9 cells were infected with the recombinant baculovirus at a 1 to 100 volume ratio, and expression cells were collected after 2 to 3 days of incubation.

The insect cell membranes were broken down through dounce homogenization in a buffer containing 25 mM HEPES (pH 7.5), 20 mM KCl, 10 mM MgCl_2_, and a protease inhibitor cocktail tablet (Sigma-Aldrich). The cell membranes were then subjected to low salt washing through centrifugation for 20 min at least two times, followed by high salt washing with a buffer containing 25 mM HEPES (pH 7.5), 20 mM KCl, 10 mM MgCl_2_, 1 M NaCl, a protease inhibitor cocktail tablet (Sigma-Aldrich), and benzonase nuclease (Novagen). This high salt washing was also conducted through centrifugation for 25 min at least two times. The purified membrane was mixed with 50% (v/v) glycerol, flash-frozen with liquid nitrogen, and stored at −80 °C until use.

The sample after membrane preparation was reconstituted with 11-*cis*-retinal overnight to reach a final concentration of 30 μM. It was then solubilized using a buffer containing 50 mM HEPES (pH 7.0), 150 mM NaCl, and 1% (w/v) n-dodecyl-β-D-maltoside (DDM). The lysate was centrifuged at 100,000*g* for 40 min at 4 °C, and the supernatant was purified through immunoaffinity chromatography using 1D4 antibody bound to CNBr-activated agarose. The 1D4 resin was added to the supernatant, mixed for 5 h, transferred to a column, and washed with a buffer containing 50 mM HEPES (pH 7.0), 150 mM NaCl, and 0.02% DDM. The purified JelRh sample was then extracted with a buffer containing 50 mM HEPES (pH 7.0), 150 mM NaCl, 0.02% DDM, and 0.2 mg/ml 1D4 peptide.

### Sample reconstitution for spectroscopy

The purified sample was reconstituted into phosphatidylcholine (PC) liposomes using a buffer containing 50 mM HEPES, 140 mM NaCl, 0.75% CHAPS, and 1 mg/ml PC at a protein–lipid molar ratio of 1:50. Dialysis was performed in order to remove DDM and the reconstituted sample was then suspended in buffer containing 2 mM NaH_2_PO_4_ (pH 7.25) and 2 mM NaCl.

### Low-temperature UV–visible and FTIR spectroscopies

The reconstituted sample was placed on a BaF_2_ window and allowed to dry naturally overnight at 4 °C before being dried further using an aspirator. A 0.5 to 1.0 uL aliquot of either H_2_O, D_2_O, or D_2_^18^O was placed next to the film for humidification, and the samples were sealed using another BaF_2_ window with the help of a silicon rubber O-ring, resulting in an optical path length of 0.5 mm.

Low-temperature UV–visible and FTIR absorption spectroscopies were performed on hydrated films using the Oxford cryostat (OptistatDN) with an Oxford temperature controller (ITC4) and liquid nitrogen as a coolant. To form the Batho intermediate, the sample was illuminated with 500 nm light (using an interference filter) for 2 min at 77 K. To revert the Batho intermediate back to the resting state, the sample was illuminated with light >610 nm (using a R63 cut-off filter) for 1 min at 77 K. For Batho to Iso-rhodopsin (9-*cis*-retinal bound resting state) conversion, the sample was illuminated with light >520 nm (using a O54 cut-off filter) for 1 min at 77 K. The difference spectra were calculated from the spectra before and after illumination. For each FTIR measurement, 128 interferograms were recorded and 80, 100, and 120 recordings (for Batho) or 100 recordings (for Iso) in H_2_O, D_2_O, and D_2_^18^O hydration were averaged, respectively. The FTIR spectra were recorded at 2 cm^−1^ resolution.

### Hydroxylamine bleach reaction

Hydroxylamine at a final concentration of 10 mM was added to the supernatant after solubilization with DDM and incubated on ice for 30 min. The sample was illuminated with >500 nm light (by using a Y52 cut-off filter) for 2 min at 10 °C. UV-visible spectroscopy was measured for the sample before and after illumination, followed by calculation the light-induced difference absorption spectra.

### Calculation of total area of HOOP vibrational bands

In light-induced FTIR difference spectra, each peak appearing in the 1000–800 cm^−1^ region corresponds to HOOP vibrational band. The area under the characteristic peak was calculated by multiplying the average height with data points using the Igor Pro-8 software.

## Data availability

The data supporting the findings of this study are available in the article, Supporting information, and if applicable, from the corresponding author on request. In addition, all the data supporting the findings of this manuscript have been deposited in Figshare.com (10.6084/m9.figshare.22313617).

## Supporting information

This article contains supporting information (64, 65, 66).

## Conflicts of interest

All authors declare that they have no conflicts of interest.
