# Supplementary material for: Investigating the mechanism of photoisomerization in jellyfish rhodopsin with the counterion at an atypical position
Source: J Biol Chem. 2023 Apr 23;299(6):104726. doi: 10.1016/j.jbc.2023.104726 (PMC10220492; doi:10.1016/j.jbc.2023.104726)
Supplement: Supporting information [file mmc1.docx]

**SUPPLEMENTARY INFORMATION**

**Investigating the Mechanism of Photoisomerization in Jellyfish Rhodopsin with the**

**Counterion at an Atypical Position**

Shino Inukai,^1^ Kota Katayama,^1,2,3*^ Mitsumasa Koyanagi,^4^ Akihisa Terakita,^4^ and Hideki Kandori^1,2*^

^1^Department of Life Science and Applied Chemistry, Nagoya Institute of Technology, Showa-ku, Nagoya 466-8555, Japan

^2^OptoBioTechnology Research Center, Nagoya Institute of Technology,

Showa-ku, Nagoya 466-8555, Japan

^3^PRESTO, Japan Science and Technology Agency,

4-1-8 Honcho, Kawaguchi, Saitama 332-0012, Japan

^4^Department of Biology, Graduate School of Science, Osaka Metropolitan University, 3-3-138, Sugimoto, Sumiyoshi-ku, Osaka 558-8585, Japan

^*^To whom correspondence may be addressed.

Email: katayama.kota@nitech.ac.jp or kandori@nitech.ac.jp

Running title: Photoisomerization mechanism of jellyfish rhodopsin

Keywords: animal rhodopsin, jellyfish, counterion, isomerization, FTIR

**Extended information:**

Figure S2 confirms that the mirror image of the spectra was also identified in the FTIR measurement by utilizing the light illumination conditions from the UV-visible spectroscopy. Additionally, Figure S3 illustrates the C-C stretching and hydrogen out-of-plane (HOOP) vibration regions in the FTIR difference spectra obtained through the light illumination dependence experiment. The 11-*cis* and 9-*cis* bound forms have distinct vibrational bands located around the 1240 cm^-1^ and 1250/1210 cm^-1^ regions for C-C stretching vibration (29, 30, 66) and around the 970 cm^-1^ and 950 cm^-1^ regions for HOOP vibration (67, 68), respectively. By utilizing these bands as markers, we aim to establish the illumination conditions for obtaining pure 11-*cis* and 9-*cis* bound spectra. Figure S3's FTIR difference spectra upon illumination at >610 nm against Batho state (red line) clearly shows the disappearance of the 1246 cm^-1^ and 962 cm^-1^ bands from the possible 9-*cis* bound form, resulting in a pure 11-*cis* bound spectra. In contrast, light illumination at >520 nm eliminated the 1236 cm^-1^ and 973 cm^-1^ bands from the possible 11-*cis* form, resulting in a spectra with the highest accumulation of the 9-*cis* form (orange line). Notably, these illumination conditions are the same as those used for low-temperature UV-visible spectroscopy in Figure 2. Figure S4 represents Batho -minus- rhodopsin (a), Batho -minus- Iso (b), and Iso -minus- rhodopsin (c) difference spectra of JelRh in H_2_O (solid line) and D_2_O (dotted line), where the Iso -minus- rhodopsin spectra was calculated by subtracting (a) from (b). The two positive and one negative peaks at 1246, 1209, and 1236 cm^-1^, respectively, are characteristic of fingerprint vibrations of 9-*cis* and 11-*cis* isomers, respectively. Similarly, the peaks at 962 and 971 cm^-1^ would correspond to HOOP vibrations of 9-*cis* and 11-*cis* isomers, respectively. Thus, the clearly identified 11-*cis* and 9-*cis*-retinal specific vibrational bands imply optimized light illumination conditions.

**References:**

[66] Kandori, H., and Maeda, A. (1995) FTIR spectroscopy reveals microscopic structural changes of the protein around the rhodopsin chromophore upon photoisomerization. *Biochemistry* **34**, 14220-14229.

[67] Eyring, G., Curry, B., Broek, A., Lugtenburg, J., and Mathies, R. (1982) Assignment and interpretation of hydrogen out-of-plane vibrations in the resonance Raman spectra of rhodopsin and bathorhodopsin. *Biochemistry* **21**, 384-393.

[68] Bagley, K. A., Balogh-Nair, V., Croteau, A. A., Dollinger, G., Ebrey, T. G., Eisenstein, L., *et al*. (1985) Fourier-transform infrared difference spectroscopy of rhodopsin and its photoproducts at low temperature. *Biochemistry* **24**, 6055-6071.


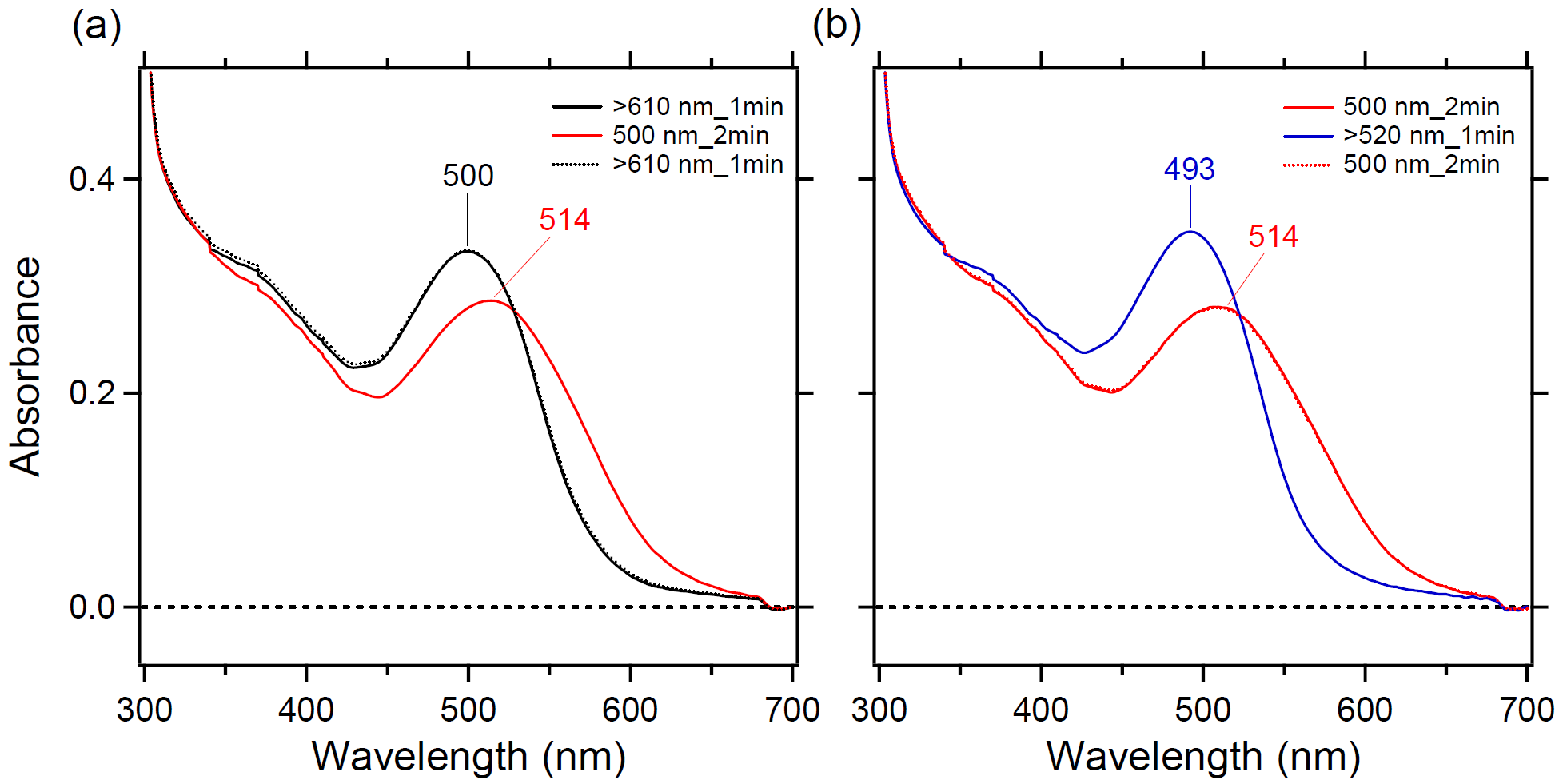


**Figure S1.** (a) UV-visible absorption spectra of Box jellyfish rhodopsin (JelRh) (black solid line) at 77 K, and primary photointermediate state, Batho having λ_max_ at 514 nm formed upon illumination at 500 nm (red line). Subsequent illumination at >610 nm causes revert photoreaction to the resting state (back dotted line). (b) UV-visible absorption spectrum of 9-*cis* bound form in JelRh (so called Iso-JelRh, blue line) having λ_max_ at 493 nm by illumination at >520 nm against Batho state spectrum (red solid line). Subsequent illumination at 500 nm shows revert photoreaction to the Batho state (red dotted line), representing the photoequilibrium between 9-*cis* and all-*trans* bound states.


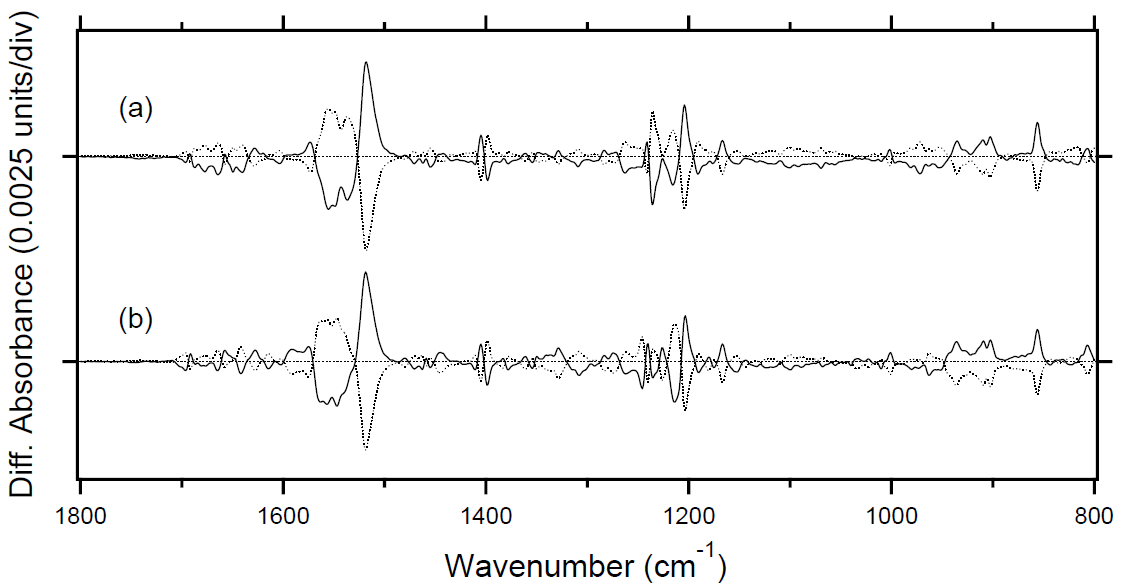


**Figure S2.** (a) Light-induced FTIR difference spectra between 11-*cis* (negative bands) and all-*trans* (positive bands) bound forms in JelRh by illumination at 500 nm, 77 K (black solid line). Subsequent illumination at >610 nm shows revert photoreaction to the resting state, resulting in the completely mirror image spectra (black dotted line). (b) Light-induced FTIR difference spectra between 9-*cis* (negative bands) and all-*trans* (positive bands) bound forms in JelRh by illumination at 500 nm for forward (black solid line) and at >520 nm for revert (black dotted line) photoreaction, respectively. Consequently, both spectra exhibit mirror image, indicating the photochromism. One division of the y-axis corresponds to 0.0025 absorption unit.


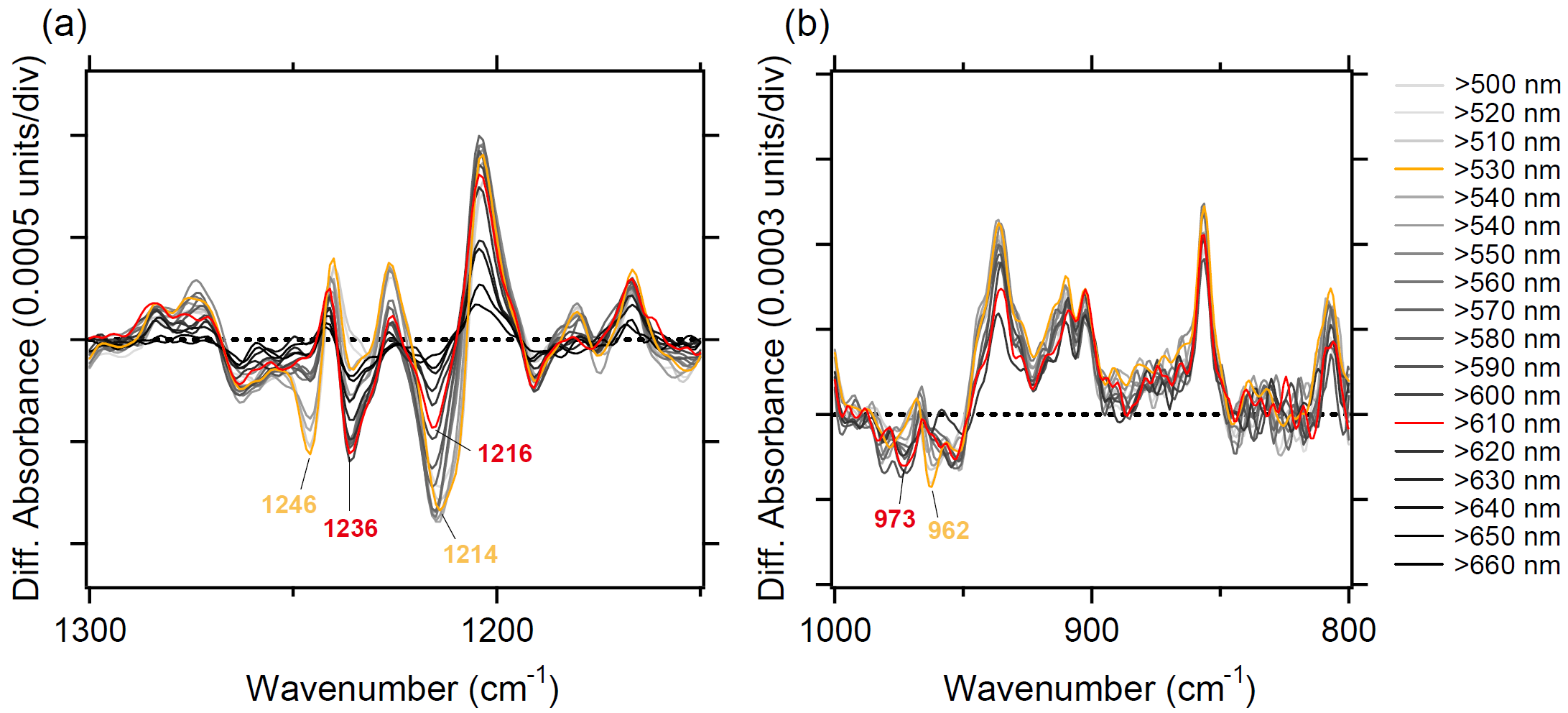


**Figure S3.** (a) Light-induced FTIR difference spectra of JelRh at 77 K by different illumination conditions from >500 nm illumination to >660 nm illumination in the 1300-1150 cm^-1^ (a) and 1000-800 cm^-1^ (b) regions, respectively. Red and orange tags correspond to the characteristic bands for 11-*cis* and 9-*cis* bound forms, respectively. One division of the y-axis corresponds to 0.0005 (a) and 0.0003 (b) absorption units.


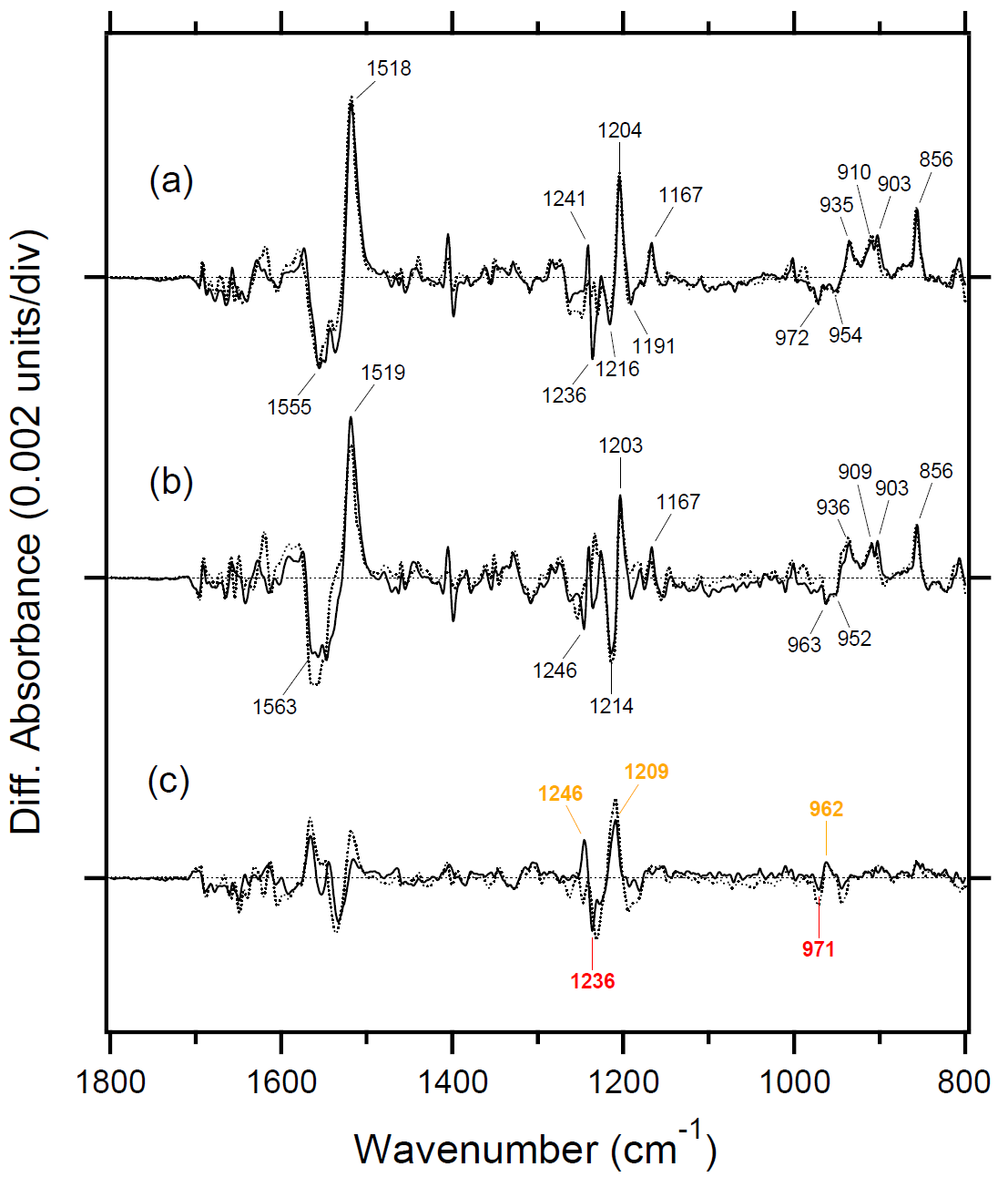


**Figure S4.** Batho -minus- rhodopsin (a), Batho -minus- Iso (b), and Iso -minus- rhodopsin (c) difference spectra of JelRh in the 1800-800 cm^-1^ region. Solid and dotted lines correspond to the spectra obtained in H_2_O and D_2_O hydrations, respectively. Batho -minus- rhodopsin and Batho -minus- Iso spectra are taken from Figure S2, and Iso -minus- rhodopsin spectra is obtained by subtracting between Batho -minus- Iso and Batho -minus- rhodopsin spectra, meaning that positive and negative bands reflect to the 9-*cis* and 11-*cis* bound forms, respectively. One division of the y-axis corresponds to 0.002 absorption unit.


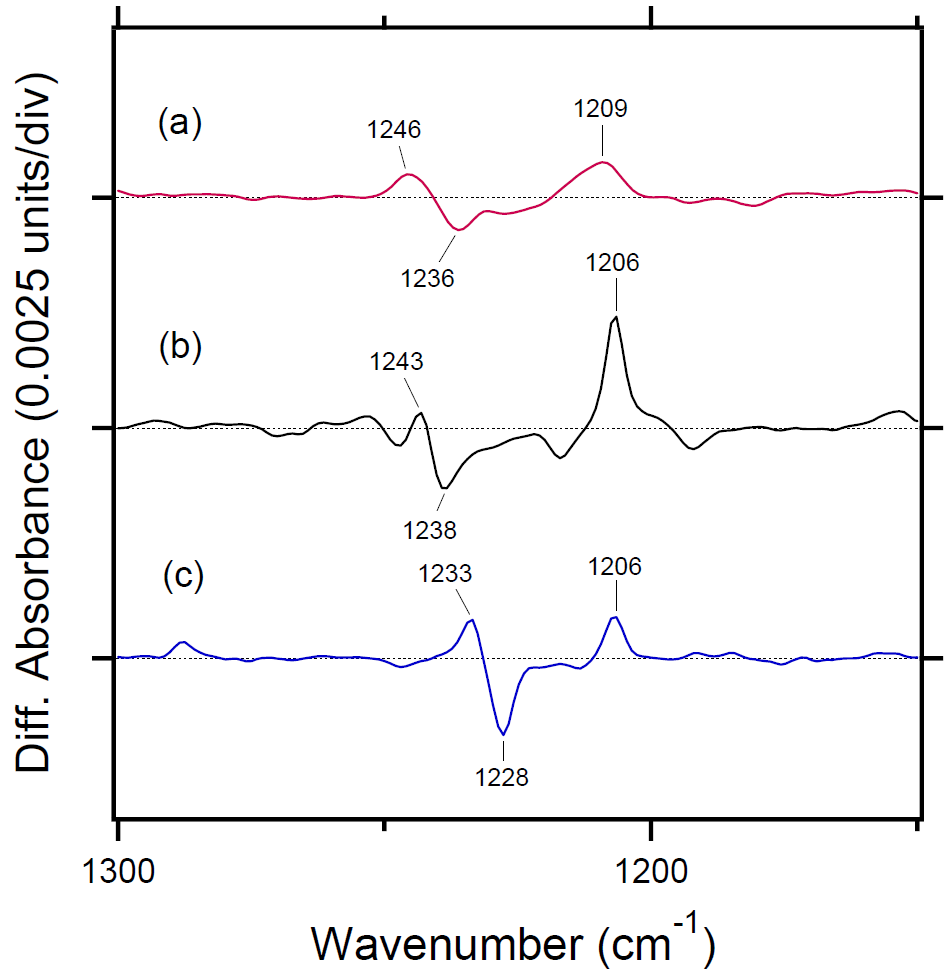


**Figure S5.** Iso -minus- rhodopsin difference spectra of JelRh (a), bovine rhodopsin (BovRh) (b), and squid rhodopsin (SquRh) (c) in the 1300-1150 cm^-1^ region, respectively. Spectra of BovRh and SquRh are taken from Ref 12 and 28, respectively. One division of the y-axis corresponds to 0.002 absorption unit. The obtained spectra of JelRh, BovRh, and SquRh are normalized by 1.0, 0.1, and 1.0, respectively.


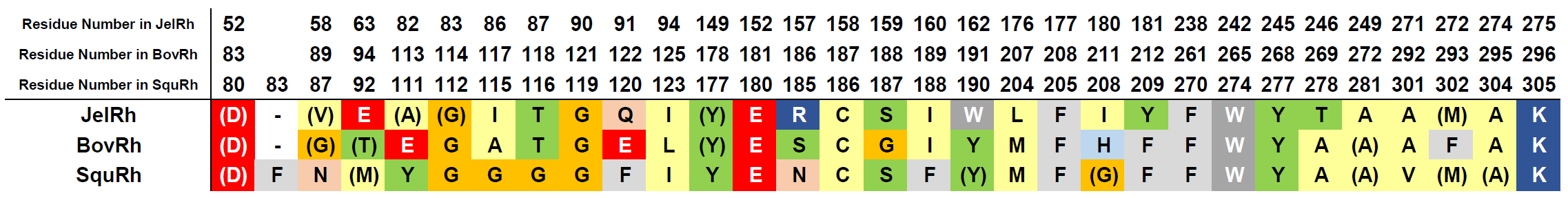


**Figure S6.** Comparison of amino acid residues between JelRh, BovRh, and SquRh within 5 Å of retinal chromophore in the crystal structure of BovRh (PDBID: 1U19 (11)) and SquRh (PDBID: 2Z73 (16)). The amino acid residues in parenthesis for JelRh and BovRh are located >5 Å of retinal chromophore in the crystal structure of BovRh, while the amino acid residues in parenthesis for SquRh are located >5 Å of retinal chromophore in the crystal structure of SquRh.


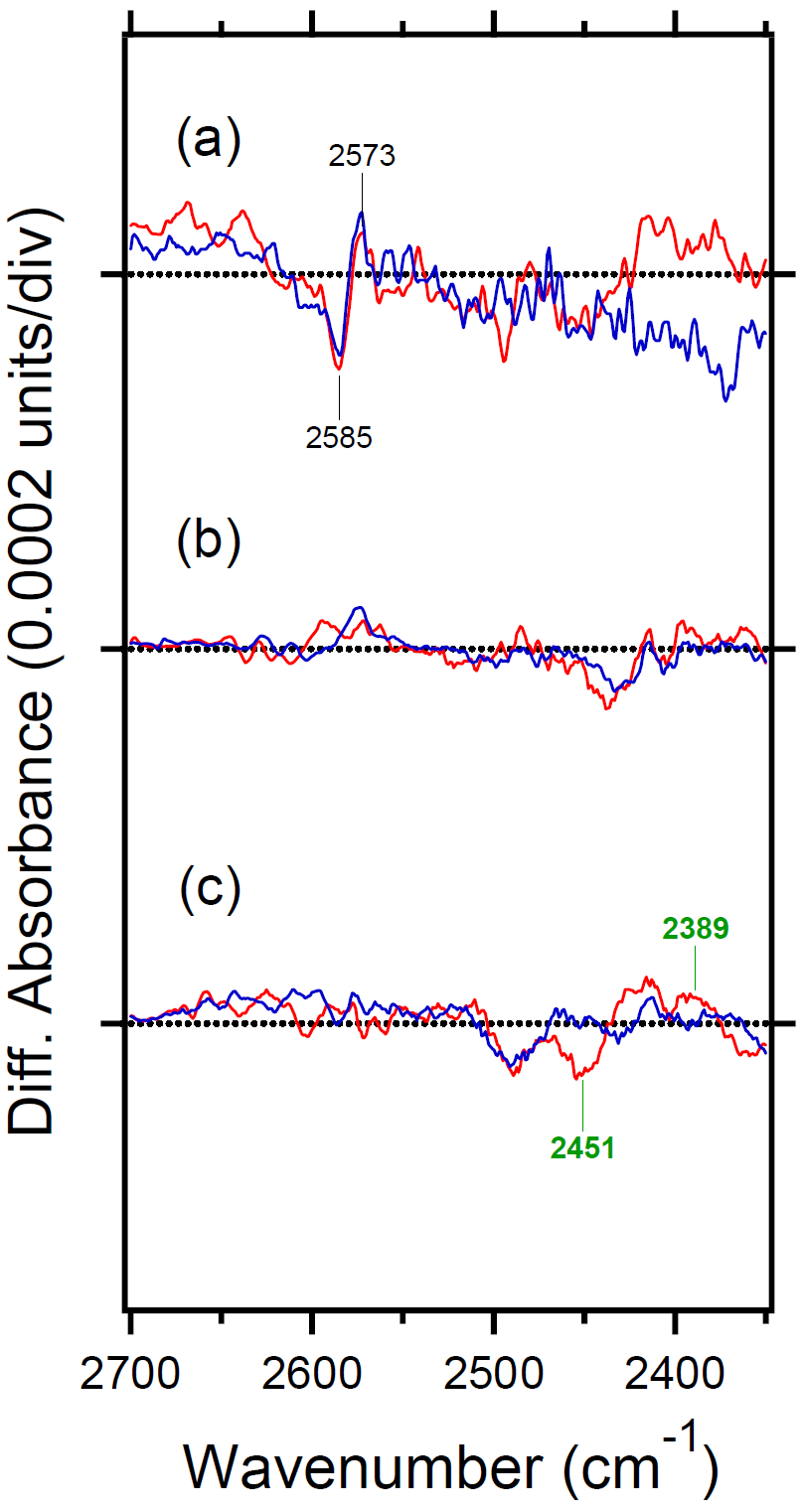


**Figure S7.** Iso -minus- rhodopsin difference spectra of JelRh (a), bovine rhodopsin (BovRh) (b), and squid rhodopsin (SquRh) (c) in the 2700-2450 cm^-1^ region, respectively. Spectra of BovRh and SquRh are taken from Ref 12 and 28, respectively. Red and blue lines are measured in D_2_O and D_2_^18^O hydrations, respectively. One division of the y-axis corresponds to 0.0002 absorption unit. The obtained spectra of JelRh, BovRh, and SquRh are normalized by 1.0, 0.1, and 1.0, respectively. The green-tagged bands exhibit the isotope effect by ^18^O and is attributed as the O-D stretching vibration of protein-bound water.


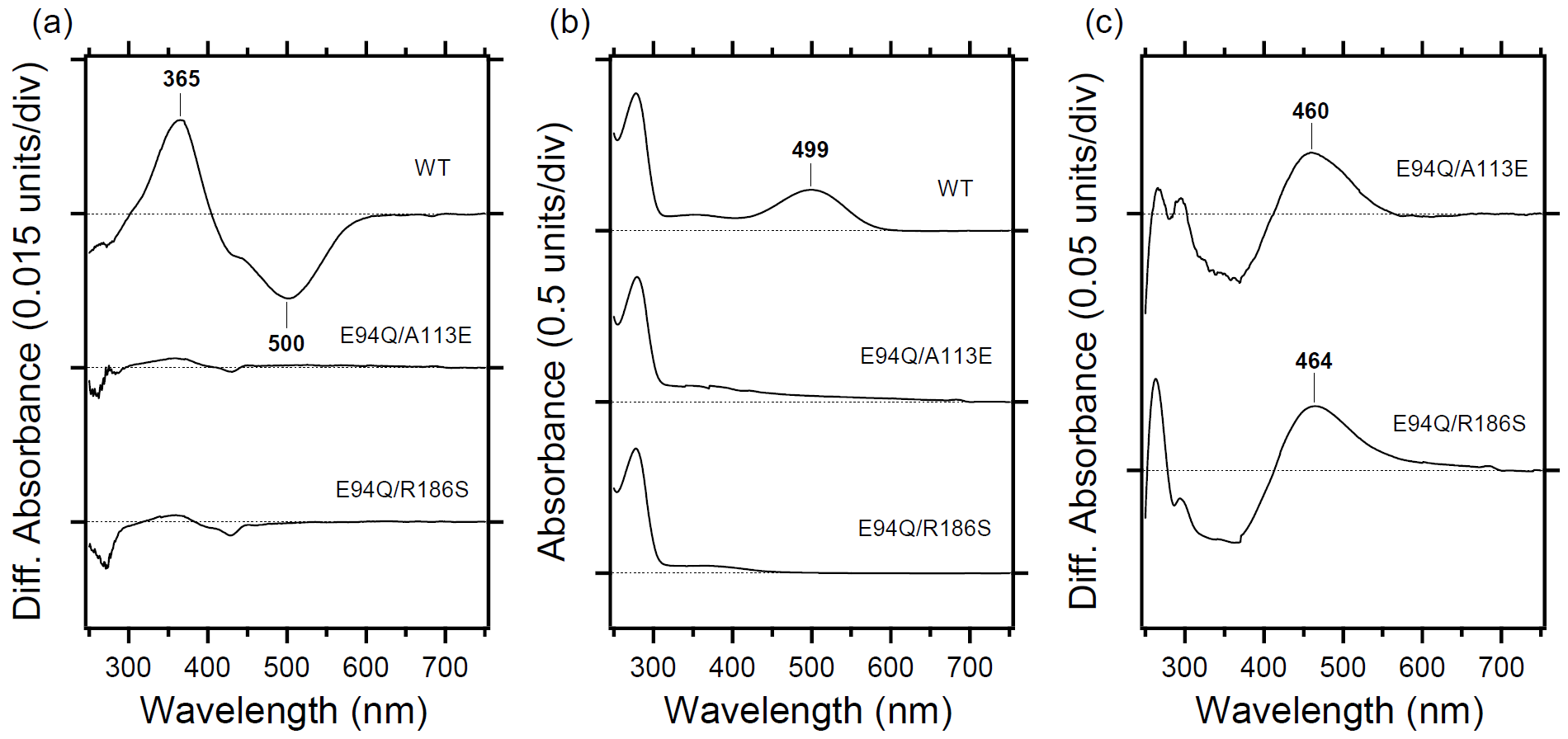


**Figure S8.** (a) Light-induced difference absorption spectra of wild-type (upper), E94Q/A113E (middle), and E94Q/R186S (bottom) of JelRh solubilized with DDM from Sf9 membrane, in the presence of 10 mM hydroxylamine. Positive and negative absorption signals show the spectra before and after illuminations, corresponding to those of the rhodopsin and the retinal oxime, respectively. (b) UV-visible absorption spectra of wild-type (upper), E94Q/A113E (middle), and E94Q/R186S (bottom) of purified JelRh in DDM solution. (c) Difference in spectrum of acidified samples (upper: E94Q/A113E, bottom: E94Q/R186S) indicate that the Schiff bases are in the equilibrium of protonated and deprotonated states.


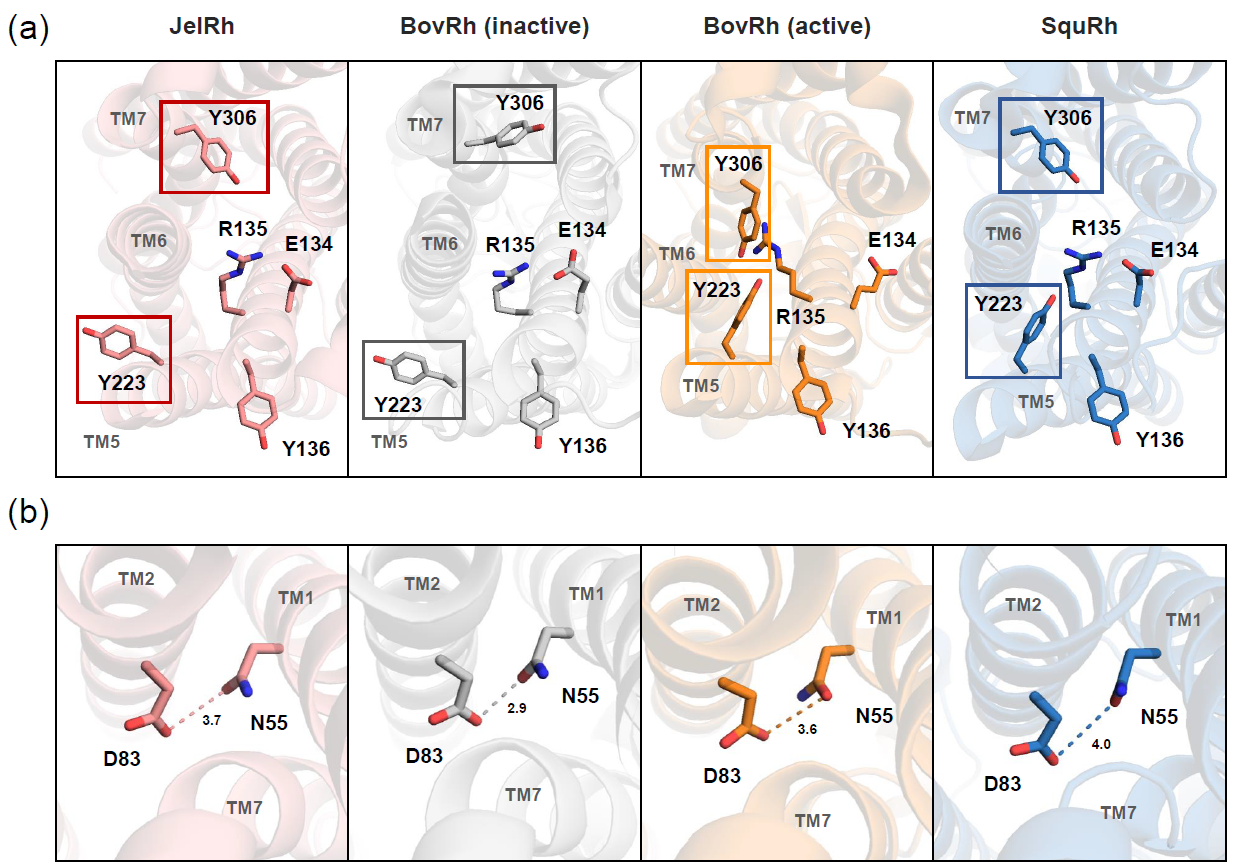


**Figure S9.** (a) Structural comparison at the cytoplasmic region between JelRh, BovRh (resting state, PDBID: 1U19 (11)), BovRh (active Meta-II state, PDBID: 3PXO (59)), and SquRh (resting state, PDBID: 2Z73 (16)) is shown. The Alpha Fold2 3D model of JelRh represents an intermediate conformation between the inactive (resting) and active states of BovRh. (i) Tyr306, NPxxY motif in TM7 points towards the TM core domain including the ERY motif, which is composed of Glu134, Arg135, and Tyr136, and is similar to the BovRh active conformation. (ii) Tyr223 in TM5 is oriented away from the TM bundle, which is most likely close to the BovRh resting conformation. (b) Structural comparison at the center of the TM bundle region between JelRh, BovRh (resting state, PDBID: 1U19 (11)), and BovRh (active Meta-II state, PDBID: 3PXO (59)) is shown. The distance between Asp83 in TM2 and Asn55 in TM1 on the Alpha Fold2 model of JelRh is 3.7 Å, which is similar to that of BovRh (active state) or SquRh (resting state), rather than that of BovRh (resting state).


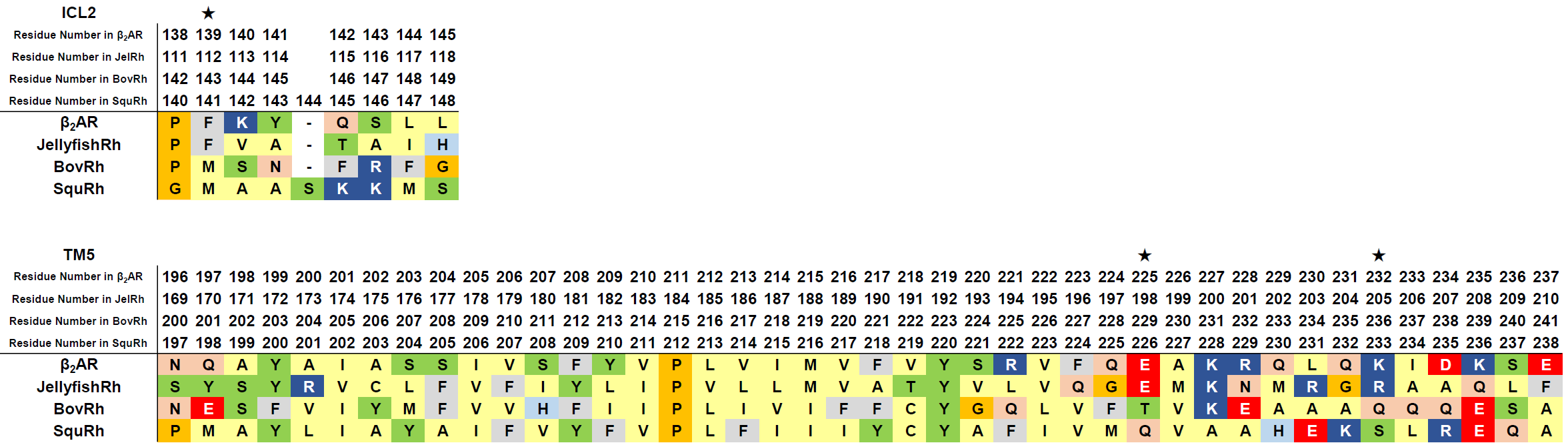


**Figure S10.** Comparison of amino acid residues between β_2_AR, JelRh, BovRh, and SquRh in ICL2 and TM5 from the crystal structure of BovRh (PDBID: 1U19 (11)). The marked amino acid residues are crucial for G_s_ coupling.
